# Serological study of vaccinia virus reservoirs in areas with and without official reports of outbreaks in cattle and humans in São Paulo, Brazil

**DOI:** 10.1007/s00705-013-1740-5

**Published:** 2013-06-13

**Authors:** Marina Gea Peres, Thais Silva Bacchiega, Camila Michele Appolinário, Acácia Ferreira Vicente, Susan Dora Allendorf, João Marcelo Azevedo Paula Antunes, Sabrina Almeida Moreira, Emerson Legatti, Clóvis Rinaldo Fonseca, Edviges Maristela Pituco, Liria Hiromi Okuda, José Carlos de Figueiredo Pantoja, Fernando Ferreira, Jane Megid

**Affiliations:** 1 Faculdade de Medicina Veterinária e Zootecnia da Universidade Estadual Paulista, Júlio de Mesquita Filho, Botucatu, São Paulo Brazil; 2 Laboratório de Viroses dos Bovídeos do, Instituto Biológico de São Paulo, São Paulo, Brazil; 3 Faculdade de Medicina Veterinária e Zootecnia da, Universidade de São Paulo, São Paulo, Brazil

## Abstract

Vaccinia virus (VACV), the etiological agent of an exanthematic disease, has been associated with several bovine outbreaks in Brazil since the end of the global vaccination campaign against smallpox. It was previously believed that the vaccine virus used for the WHO global campaign had adapted to an unknown wild reservoir and was sporadically re-emerging in outbreaks in cattle and milkers. At present, it is known that Brazilian VACV is phylogenetically different from the vaccinia virus vaccinal strain, but its origin remains unknown. This study assessed the seroprevalence of orthopoxviruses in domestic and wild animals and farmers from 47 farms in three cities in the southwest region of the state of São Paulo with or without official reports of outbreaks in cattle or humans. Our data indicate a low seroprevalence of antibodies in wild animals and raise interesting questions about the real potential of wild rodents and marsupials as VACV reservoirs, suggesting other routes through which VACV can be spread.

## Introduction

Vaccinia virus (VACV), the prototype of the genus *Orthopoxvirus* (OPV), is the etiological agent of an exanthematic disease characterized by cutaneous lesions in cow udders and teats. The disease causes economic losses due to reduction in milk production and increased susceptibility to mastitis and secondary bacterial infections. VACV is a zoonotic disease, and viral transmission occurs mainly through direct contact between milkers and cattle [1, 2]. Since the end of the global vaccination campaign against smallpox, several VACV outbreaks affecting both dairy cattle and milkers have been reported in Brazil. The first reported outbreaks occurred in the city of Cantagalo in Rio de Janeiro State and the city of Araçatuba in the state of São Paulo. These isolated viruses were named Cantagalo virus (CTGV) and Araçatuba virus (ARAV), respectively [3–6].

The largest milk-producing state in Brazil, Minas Gerais, has reported outbreaks affecting cattle and milkers in the last 10 years [7]. In the state of São Paulo, in Torre de Pedra, Guareí and Itatinga Counties, outbreaks have been reported affecting cows and humans since 2007 [8, 9]. Other outbreaks have been reported in the states of Mato Grosso and Rondonia; however, the origin of VACV remains unknown [10]. It was previously believed that the vaccinia vaccinal virus of the WHO smallpox global campaign, particularly VACV-IOC, had adapted to an unknown wild reservoir and was sporadically re-emerging by means of outbreaks in cattle and milkers. This was inferred because genetic studies demonstrated that VACV-IOC displays the same deletion in the A56R gene as some Brazilian VACV isolates. In addition, these same studies also demonstrated that some nucleotide substitutions present in the vaccine virus are not shared by Brazilian VACV. Therefore, the most widely accepted theory is that there are genetically and phenotypically different VACV populations circulating in unknown natural reservoirs, and the origin of the virus remains unknown. The transmission of these VACV strains to cows and humans depends on biological and geographical conditions [6, 11, 12].

In the present study, we assessed the seroprevalence of OPV in cows, horses, sheep, swine, dogs, cats and wild specimens from the orders of Marsupialia, Carnivora and Rodentia, as well as rural workers, milkers and their families on 47 farms throughout three cities in the southwestern region of the state of São Paulo with or without a history of outbreaks. Our data indicate the low seroprevalence of antibodies in wild animals and raises interesting questions regarding the real potential of wild rodents and marsupials as reservoirs in addition to suggesting other routes of viral environmental spread.

## Materials and methods

This study was approved by the Ethical Committee of Animals Uses in Veterinary Medicine and Animal Production of São Paulo State University “Júlio de Mesquita Filho” (number 112/2010-CEUA) and by the Ethical Committee of Medicine of that university (number CEP3605-2010).

### Epidemiological survey data

To investigate each farm, epidemiological data were collected. The risk factors analyzed were as follows: milking type, presence of domestic mammals (cats, dogs, horses, swine, and sheep), problems with flies and/or ticks affecting herds, presence of synanthropic rodents and bats in the common areas of farms, presence and contact with wild animals in the peridomestic area, source of water, sewage system, garbage destination, history of previous outbreaks affecting cattle and humans, and age of rural workers and their families. Data collection was conducted from October to December 2010.

### Site sampling

**Fig. 1:**
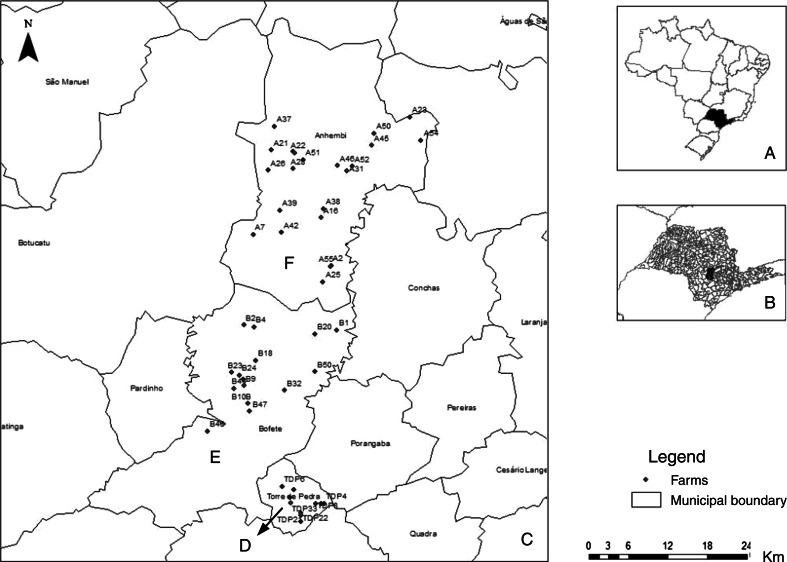
Map of sampling sites in Brazil (**a**) with São Paulo State in black. São Paulo state map (**b**) with Torre de Pedra, Bofete and Anhembi in red. Map of São Paulo State (**c**) showing the sites of sampling; the points in red correspond to farms in Torre de Pedra (**d**), Bofete (**e**) and Anhembi (**f**)

Samples were collected in three counties with and without a history of outbreaks in cattle and humans: Torre de Pedra (23^o^14’58.76’’S48^o^11’39.49’’W), where outbreaks were registered in 2007 and 2010 [8, 9], Bofete (23^o^05’54.51’’S48^o^11’26.61’’W), and Anhembi (22^o^47’09.11’’S48^o^07’30.90’’W). The latter two counties had no history of outbreak reports (Fig. 1).

The total number of farms included in the study (48) was calculated based on the population of the farms in the region (Anhembi, Bofete, and Torre de Pedra), a 5 % prevalence of positive farms (at least one positive sample), and a 5 % margin of error using Epi Info 3.5.4 [13]. Forty-eight properties were selected randomly by lot, with 11 properties being in Torre de Pedra, 15 in Bofete, and 22 in Anhembi (Fig. 1). All owners signed an informed consent form before the initiation of sample collection.

### Collection of samples from domestic animals

For each farm, the minimum number of sampled animals was determined using the program HERDACC3.0^®^ [14], assuming a PCR sensitivity of 80 %, a specificity of 99.9 %, and a proportion of infected animals in a positive herd of 20 %. After considering the variation of the herd size and the positive cutoff, the simulations determined that the minimum number of cows that needed to be sampled to ensure a minimum sensitivity and specificity of 95 % was 20 animals.

In a cattle herd with more than 20 animals, only 20 samples were collected, but in a herd with fewer than 20 animals, samples from were collected all of the animals. For other species (horses, sheep, swine, dogs and cats), from one to five samples were collected for each species. Blood samples were collected by mammary vein puncture or jugular puncture and later centrifuged, and the sera were stored at −20 °C. These samples were collected from February to April 2011.

### Collection of samples from wild animals

Capture of wild animals was conducted from May to September 2011. This work was approved by the Environment Ministry (MMA), the Brazilian Institute of Environment and Natural Resources Renewable (IBAMA), the Chico Mendes Biodiversity Conservation Institute (ICMBio), and the Biodiversity Information and Authorization System (SISBIO) for wild mammal and rodent capture under the authorization number 23918-1.

Tomahawk traps were used for capture of wild mammals using chicken bait. Wild rodents were captured with pitfall traps and Sherman traps. Peanut cream, canned sardines, cornmeal and oatmeal were used as baits. The mammals were anesthetized with tiletamine and zolazepam (Zoletil^®^) using the recommended dose for each species [15], and blood samples were collected by jugular or cardiac puncture. The animals were put inside the Tomahawk until the cessation of the anesthesia and were released the next morning. During the checking procedure for the Sherman and pitfall traps, a positive pressure mask with a HEPA filter and a triple layer of gloves were used [16]. If a rodent was present in the pitfall trap, the animal was removed and placed in a plastic box for transport to the site of sample collection. Sherman traps containing rodents were transported to the site of sample collection. The transportation of animals and traps was performed using rodent-related infectious disease control safety procedures to prevent, for instance, *Hantavirus* infection [16]. The materials used during the collection of the samples and the traps were disinfected with a benzalkonium chloride solution [16].

To collect rodent samples, the researchers used personal protective equipment (PPE) consisting of a waterproof polypropylene disposable apron, two pairs of procedure gloves, rubber boots and a respiratory motorized breathing mask made of Tyvek and including a trachea, motor and HEPA filter [16]. The rodents were anesthetized in a plastic autoclavable bag containing gauze soaked in ethyl ether. After sedation, the animals were weighed, and blood samples were collected by cardiac puncture. After this procedure, the rodents were euthanized by increasing the anesthetic and their organs were collected, placed into microtubes and stored at −80 °C for future PCR assays.

### Human sample collection

Blood samples from the farmers, rural workers and their families were collected by nurses by cephalic vein puncture during October and November 2011. The blood samples were centrifuged, and the sera were stored at −20 °C for serological study.

### Virus neutralization test

Serum from a bovine infected with the VACV Araçatuba strain collected at day 20 pi and fetal bovine serum were used as positive and negative controls, respectively, in a standard virus neutralizing (VN) assay. Test sera including a negative and a positive control were inactivated by heating at 56 °C for 30 minutes and serially diluted, starting at 1:2 in Eagle’s/HEPES containing antibiotics. The virus neutralization (VN) assay was carried out in 96-well plates, testing twofold dilutions of sera against a fixed dose of vaccinia Araçatuba virus (100 TCID_50_ [50 % tissue culture infectious dose]). The serum-virus mixtures were incubated for 18 hours at 37 °C with 5 % CO_2,_ ensuring a sensitivity of approximately 100 % [17], and then a suspension of Vero cells (3 x 10^5^ cells/ml) was added to each well. The plates were reincubated under the same conditions and VN readings were performed after four days of incubation. The titer was defined as the reciprocal of highest dilution of serum that prevented the production of CPE in indicator Vero cells. All of the tests were performed in duplicate. Titers equal to or greater than 16 for domestic animals and humans were considered positive [18].

### Plaque-reduction neutralization test (PRNT)

Serum samples were inactivated by heating at 56 °C for 30 minutes, and later, an OPV plaque-reduction neutralization test (PRNT) was performed. Inactivated sera were diluted from 1:20 in MEM and subjected to PRNT in BSC-40 cells as described previously [17] using the VACV-Western Reserve strain. As positive controls, anti-OPV-positive human sera collected during BV outbreaks were used [19]. As negative controls, bovine anti-OPV negative sera were used. A serum sample was considered positive if it caused at least a 50 % reduction in the number of viral plaques compared with the negative controls (corresponding to a titer of 20) [20]. The OPV-PRNT specificity (97.4 %) and sensitivity (93.5 %) were confirmed using receiver-operating characteristic analysis, comparing results of PRNT, ELISA and clinical symptoms obtained during BV outbreaks [20].

### Statistical analysis

Descriptive analyses were performed to produce frequency distributions for the different types of samples, species, serological results, and other variables studied. The chi-square test or Fisher′s exact test [21] was used to test the association between the serological positivity for OPV and each factor studied. The aforementioned associations were further adjusted by city using the Cochran-Mantel-Haenszel method because city was a significant confounding factor.

## Results

### Missing values

The total number of farms chosen for the study was 48; however, of the 11 farms selected in Torre de Pedra, only 10 farmers authorized the study, resulting in a total of 47 farms sampled.

### Samples collected and seropositivity

Of the 1331 serum samples collected, 14 % had positive titers. The highest positivity among domestic species was observed in dogs (22.8 %), swine (18.2 %), and cows (15.3 %). In the general context, humans showed the third-highest positivity rate (17 %). The wild species sampled had a lower positivity rate, with 8.7% of wild rodents being positive, and 8.2 % of *Didelphis spp* being positive. Out of four *Nasua nasua* samples, only one tested positive (Table 1).

**Table 1:** Total samples collected and the percentage of positives by species

Species	No. of samples collected	Serological test	Positives (%)
Cow	688	VN*	105 (15.3)
Horse	117	VN	9 (7.7)
Sheep	44	VN	0 (0.0)
Swine	22	VN	4 (18.2)
Dog	114	VN	26 (22.8)
Cat	7	VN	1 (14.3)
Human	148	VN	25 (16.9)
Didelphis spp***	73	PRNT**	6 (8.2)
*Gracilinanus microtarsus*	6	PRNT	0 (0.0)
*Nasua nasua*	4	PRNT	1 (25.0)
*Cerdocyon thous*	4	PRNT	0 (0.0)
Leopardus pardalis****	1	PRNT	0 (0.0)
Wild rodents^1^	103	PRNT	9 (8.7)
Total	1331		186 (13.9)

* VN = virus neutralization test

** PRNT = plaque reduction neutralization test

*** Didelphis spp were grouped: *Didelphis albiventris* (57 samples collected; 4 positives; 7.0%) and *Didelphis aurita* (16 samples collected; 2 positives; 12.5%)

**** Leopardus pardalis was not included in the statistical analysis

^1^ Wild rodents were analyzed as one group, but the species are specified in Table 2

Of the 103 wild rodents sampled, four *Oligoryzomys nigripes,* three *Oligoryzomys flavenscens* and two *Sooretamys angouya* tested positive (Table 2). The neutralizing titers ranged from 16 to 2048 for domestic animals (n = 992) and humans (n = 148) (Table 3). In the wild species, 8.4% (n = 190) tested positive in the PRNT. Neutralizing titers were positively associated with age in humans (p < 0.0001). The highest percentage of positivity was observed within the older age categories (22 to 32 years, 10 %; 32 to 42 years, 15 %; 42 to 52 years, 23 %; 52 to 62 years, 35 %; and > 62 years old, 47 % positive).

**Table 2:** Wild rodents species sampled and their positivity for OPV

Species	No. of samples	No. positive
*Oligoryzomys nigripes*	61	4
*Oligoryzomys flavenscens*	17	3
*Calomys tener*	4	0
*Nectomys squamipes*	4	0
*Akodon montensis*	4	0
*Sooretamys angouya*	13	2

**Table 3:** Percentage of domestic animals and humans with positive neutralizing antibody titers against OPV

Neutralizing titer*	Frequency	Percent (%)
16	56	4.9
32	33	2.9
64	32	2.8
128	19	1.7
156	12	1.0
512	7	0.6
1024	8	0.7
2048	3	0.3

* A VN test titer equal to or greater than 16 is considered positive for OPV

Of the total collected samples, 683 were from Anhembi, 395 from Bofete and 253 from Torre de Pedra. The proportion of total positive samples from domestic animals was not homogeneous among cities (p < 0.0001). The proportion of positive samples was not different among cities for both humans (p = 0.27) and wild animals (p = 0.88). The greatest proportion of positive samples for humans and domestic animals was found in Torre de Pedra (Table 4).

**Table 4:** Sample distribution among cities and differences in the proportion of humans and animals positive for OPV

Sample	Anhembi		Bofete		Torre de Pedra		*P-*value^1^
	n	Positive (%)	n	Positive (%)	n	Positive (%)	
Total	683	51 (7.5)^a^	395	60 (15.2)^b^	253	75 (29.6)^c^	0.0001
H*	82	12 (14.6)^a^	38	7 (18.4)^a^	28	6 (21.4)^a^	0.2778
DA**	504	31 (6.2)^a^	285	46 (16.1)^b^	203	68 (33.5)^c^	0.0001
WA***	97	8 (8.3)^a^	72	7 (10.0)^a^	22	1 (4.5)^a^	0.8711

H*, humans; DA**, domestic animals; WA***, wild animals

^1^Proportions with the same superscript within the same row are not different

Regardless of the city, the proportion of positive samples was different between domestic and wild animals (p < 0.0583), and there were more domestic than wild positive animals (Table 4). Similarly, regardless of the city, the proportion of positive samples between domestic species was different (p < 0.0084), but the proportion was not different among wild species (p = 0.6962). Torre de Pedra had the highest positivity in cows (39 %) and horses (22 %). Bofete had the highest positivity in dogs (36 %) and the second-highest positivity in cows (14.2 %) and horses (9 %). Anhembi had the lowest positivity in domestic species. Considering the wild species, Torre de Pedra had the highest positivity in *Didelphis spp* (10%), Bofete exhibited the highest positivity in wild rodents (11 %), and Anhembi demonstrated the highest positivity in *Nasua nasua* (33 %) (Table 5).

**Table 5:** Differences in the proportion of samples positive for OPV between domestic and wild species

	Anhembi		Bofete		Torre de Pedra	
	n	Positive (%)	n	Positive (%)	n	Positive (%)
Domestic						
Cow	332	17 (5.1)	204	29 (14.2)	152	59 (38.8)
Horse	72	2 (2.8)	22	2 (9.0)	23	5 (21.7)
Sheep	33	0 (0.0)	9	0 (0.0)	2	0 (0.0)
Swine	9	1 (11.1)	12	2 (16.7)	1	1 (100.0)
Dog	55	11 (20.0)	36	13 (36.1)	23	2 (8.7)
Cat	3	0 (00)	2	0 (0.0)	2	1 (50.0)
Wild						
*C. thous*	3	0 (0.0)	1	0 (0.0)	0	
*N. nasua*	3	1 (33.3)	1	0 (0.0)	0	
*G. microtarsus*	2	0 (0.0)	2	0 (0.0)	2	0 (0.0)
*Didelphis spp*	31	2 (6.5)	32	3 (9.4)	10	1 (10.0)
Wild rodents	57	5 (8.8)	36	4 (11.1)	10	0 (0.0)

### Epidemiological data – association between risk factors and positivity

The survey indicated that 96 % of farmers declared that their domestic animals have contact with wild animals, and 81 % reported the presence of rodents and bats in the farm common areas. Regarding problems with flies and ticks, 90 % reported problems with flies, and 94 % said they had problems with ticks. In addition, 22 % reported not having a sewage system, 74 % declared owning a cesspit, 2 % declared having access to a public sewage treatment system and 1 % declared owning two of these sewage treatment systems. Moreover, 75 % reported taking garbage to a public collection site, while 25 % reported burning or burying trash on their farm. Regarding water sources, 72 % reported having a headspring, 24 % reported owning a well, and 4% reported having access to a public water supply. Seventy-five percent of farmers reported manual milking, and 25 % reported using mechanical milking systems. Concerning the history of outbreaks affecting cattle and humans, 54 % said VACV had affected animals in the herd, and 17 % also reported encountering the disease in humans.

There was no association between positivity in cows and the type of milking based on city (p = 0.5662), nor between the positivity of humans and the type of milking (p = 0.5216). The associations between all of the risk factors analyzed regardless of the city and positivity were verified against history of outbreaks affecting humans, history of outbreaks affecting cattle, source of water, and destination of garbage. The history of outbreaks affecting humans was associated with positivity only for humans (p = 0.02). A history of outbreaks affecting cattle was associated with positivity in humans (p = 0.00) and domestic animals (p = 0.00), but there was no association with positive test results for wild animals (p = 0.60). The source of water was associated with positivity only for humans (p = 0.02). Garbage destination was another factor associated with positivity in domestic animals (p = 0.03) (Table 6).

**Table 6:** Risk factors for OPV infection and their association with positivity in humans, domestic animals and wild animals

Risk factor	Humans			Domestic			Wild		
	Positive (%)	n	*P -* value	Positive (%)	n	*P -* value	Positive (%)	n	*P -* value
VACV h^**1**^			0.02			0.58			0.10
Yes	2 (6.4)	31		35 (19.8)	177		3 (15.0)	20	
No	23 (19.5)	118		110 (13.5)	815		13 (7.6)	170	
VACV c^**2**^			0.00			0.00			0.60
Yes	8 (10.0)	80		73 (13.1)	557		8 (9.6)	83	
No	17 (24.6)	69		72 (16.5)	435		8 (7.5)	107	
Water			0.02			0.25			0.79
H^3^	23 (21.7)	106		102 (14.6)	699		14 (9.0)	155	
w^4^	1 (2.6)	38		28 (11.3)	248		2 (6.0)	34	
PWS^5^	1 (20.0)	5		15 (33.3)	45		0 (0.0)	1	
Garbage			0.87			0.03			0.31
PC^6^	19 (16.9)	112		127 (16.9)	748		13 (10.0)	133	
BBT^7^	6 (26.2)	37		18 (7.4)	244		3 (5.3)	57	
Sewage			0.18			0.35			0.56
No^8^	8 (24.3)	33		36 (16.2)	223		5 (12.5)	40	
C^9^	16 (14.3)	112		106 (14.5)	728		11 (7.5)	147	
PSS^10^	1 (50.0)	2		1 (3.7)	27		0 (0.0)	1	
O^11^	0 (0.0)	2		2 (14.3)	14		0 (0.0)	2	
Bats			0.15			0.66			0.26
Yes	23(18.8)	122		125(15.2)	820		13 (9.6)	135	
No	2 (7.4)	27		20 (11.6)	172		3 (5.4)	55	
Rodents			0.51			0.78			0.38
Yes	21 (17.0)	118		122(14.7)	827		10 (7.3)	137	
No	4 (12.9)	31		23 (13.9)	165		6 (11.3)	53	
Ticks			0.47			0.43			0.69
Yes	24 (17.4)	138		140(14.8)	945		15 (9.0)	167	
No	1 (9.0)	11		5 (10.6)	47		1 (4.3)	23	
Flies			0.85			0.44			0.40
Yes	22 (16.9)	130		140(15.9)	883		16 (8.7)	183	
No	3 (15.8)	19		5 (4.6)	109		0 (0.0)	7	
Wild^**12**^			0.93			0.97			0.82
Yes	24 (16.7)	144		130(13.7)	947		16 (8.4)	189	
No	1 (20.0)	5		15 (33.3)	45		0 (0.0)	1	

1) VACV h = history of outbreaks affecting humans. 2) VACV c = history of outbreaks affecting cattle. 3) H = headspring; 4) W = well; 5) PWS = public water system; 6) PC = public collection; 7) BBT = burn or bury trash on farm; 8) No = do not have a sewage system; 9) C = cesspit; 10) PSS = public sewage system treatment; 11) O = other (two of these sewage systems); 12) Wild = contact of domestic animals with wild animals

## Discussion

The circulation and maintenance of VACV in the environment has been studied in recent years in Brazil, but these conditions have not been fully elucidated, nor has the possible involvement of wild species as reservoirs [6, 22]. In this work, we analyzed the risk factors that could be associated with VACV circulation among humans, domestic animals and wildlife, and the maintenance of this virus in the environment in areas with and without officially reported outbreaks. To the best of our knowledge, this study is the first to investigate the real potential of wild rodents serving as reservoirs in their natural environment in the state of São Paulo.

Since the isolation of a supposed VACV in one wild rodent of the genus *Oryzomys* in the Amazon rain forest [23], those rodents have been identified as possible reservoirs of the virus. This idea was reinforced by experimental studies demonstrating that mice can acquire the infection through contact with the feces of experimentally infected cows, and the virus eliminated in their feces can be a source of infection for cows and sentinel mice exposed to them [12, 24]. These data provide the basis for a proposed transmission model in which peridomestic rodents act as a connection between domestic animals and wildlife in the rural environment [22]. However, the results of recent sequencing of the Cotia virus genome have shown that this virus does not belong to the genus *Orthopoxvirus*, as previously believed [25], suggesting that VACV has never been isolated from a wild rodent. Unexpectedly, our data provide evidence of a low seroprevalence of orthopoxviruses among wild rodents sampled in this region of São Paulo state, associated with a high seroprevalence found in domestic animals, which leads us to believe that *Oligoryzomis nigripes*, *Oligoryzomis flavenscens*, *Calomys tener*, *Nectomys squamipes*, *Akodon montensis* and *Sooretamys angouya* are not VACV reservoirs in this Brazilian region. In Torre de Pedra, a city where outbreaks have been reported previously [8, 9], a high seroprevalence among domestic animals and humans was detected, and there was no positive result from wild rodents, reinforcing the low probability that these species act as reservoirs in the VACV cycle.

Other wild mammal species have been suggested as VACV reservoirs, such as primates and members of the order Carnivora [20]. Our study demonstrated that the positivity in opossums (*Didelphis albiventris* or *Didelphis aurita*) was low in the three cities, and the positivity (25 %) in coati (*Nasua nasua*) was not representative because of the small sample size (of four samples, only one was positive). In this context, we suggest that if these wild animals are not involved in VACV transmission to cows, they might, conversely, acquire the virus from cows. It is know that experimentally infected cows eliminate the virus in their feces. D’Anunciação et al. [24] demonstrated that BALB/c mice exposed to feces of experimentally infected cows acquired the infection and eliminated virus in their feces, suggesting that the feces of infected bovines could represent a constant source of environmental virus contamination. Rivetti et al. [26] demonstrated that experimentally infected cows eliminate viral DNA in their feces from the first day postinfection and continue to do so even after resolution of the lesions for approximately 70 days after infection. These authors also suggested that feces from infected cows may be an important source of VACV transmission, contributing to virus dissemination among farms. This hypothesis could not be confirmed in the present study, as positivity in wild rodents were not demonstrated in Torre de Pedra, a city with two previous outbreaks in 2007 and 2010 [8, 9] and where a positivity rate of 39 % was detected in the cows sampled (Table 5). Torre de Pedra was expected to have the highest positivity of wild specimens, but our results demonstrated that Torre de Pedra had the highest positivity only for *Didelphis spp* (10 %) compared to Anhembi and Bofete. This positivity of 10 % was not significant, and in addition to the finding that no wild rodents tested positive in Torre de Pedra, we suggest that the feces of infected cows does not represent an efficient way of spreading VACV to the environment; alternatively, we suggest that the wild species studied here are not efficient VACV reservoirs. Given this finding, we suggest searching for other pathways of VACV transmission and dissemination in the environment.

The highest percentage of positivity was found in dogs, followed by decreasing values of positivity in swine, humans, cows, cats and horses. However, considering the positivity among species in the three cities, dogs had a higher percentage of positive samples only in Bofete and Anhembi, because in Torre de Pedra, as expected, the highest percentage of positive samples was found in cows, among which there had been two previous outbreaks [8, 9]. This higher percentage of positive dog samples was not expected, but it does leads us to question if dogs are involved in the process of spreading the virus or are merely accidental hosts. As there are no reports of dogs developing exanthematic lesions from VACV, and because dogs did not exhibit lesions suggestive of poxvirus infection during our sample collection, dogs may be only infected without clinical signs, or they may be a natural reservoir of the virus. The high frequency of positive titers in dogs can be explained by their close contact with humans and their livestock, with sick cows and the environment contaminated by cow excretions, being infected without clinical signs and being only an accidental host that might eventually spread VACV to the environment. Our results are still insufficient to answer such questions, but the dichotomy of VACV isolated in Brazil may be related to the absence of clinic signs in dogs, as group I (ARAV, PSTV, GP2 V,) of VACVs when inoculated intranasally in BALB/c mice does not induce clinical signs [6, 11, 12]. As demonstrated by Megid et al. [9], the VACV involved in the Torre de Pedra outbreak in 2010 was closely related to ARAV and CTGV, which are group I viruses. Another possibility to consider is a cross-reaction with another poxvirus, as serology is not specific for vaccinia virus [27]. This possibility must be considered but does not seem probable, as dogs were the third-most prevalent species in the city of Torre de Pedra, being outnumbered only by cows.

No clinical signs were observed in other domestic animals during the collection of samples. VACV outbreaks have been described in horses [28]; however, they were not reported in Torre de Pedra when outbreaks were affecting cows and humans, and according to this study, horses were the domestic species with the second-highest rate of positivity. This high positivity rate without clinical signs leads us to suppose that horses, like dogs, may be only subclinically infected, resulting in seropositivity, and they might serve as VACV reservoirs or accidental hosts. It is also possible that this VACV strain only induces clinical signs in cows and humans.

The titers of this virus in humans should be carefully evaluated. The highest percentage of positivity in domestic and wild animals was observed in Bofete and Anhembi, and the second highest positivity was observed in Torre de Pedra. Studies have associated the titers of antibodies in farmers who were born before 1977 with antibodies induced by the smallpox vaccine [7]. In this study, the humans that were born before 1977 declared that they did not know if they were vaccinated against smallpox. Our results indicated that positivity was directly correlated with age, and higher in older persons, and this leads us to believe that these antibodies could be associated with lifelong exposure of farmers to the virus, but smallpox vaccine memory cannot be excluded. Another possibility that cannot be dismissed is the association of seropositivity between dogs and humans, since dogs were the domestic species with the highest percentage of positivity. Due to their close contact with humans, dogs might not only be possible reservoirs or accidental hosts but might also be involved in transmission to or from humans. Another hypothesis is that, considering the close living between dogs and humans both have the same possibility to be exposed to virus reservoirs.

The risk factors analyzed in this study indicated, as expected, that there was an association between a history of outbreaks affecting humans and positivity in humans and between a history of outbreaks affecting cattle and positivity in humans and domestic animals. Conversely, garbage destination was associated with positivity only for domestic animals. The highest positivity of domestic animals was observed when farmers declared that they transported their garbage to a public collection site. It is likely that this association is indirectly related to the movement of humans between farms [29]. It was not possible in this study to determine the actual mechanisms involved in this association, but this risk factor should be investigated further. Overall, our results provide a new focus of possible VACV reservoirs and viral spread. In addition, these findings demonstrate that the wild species sampled in this study are not the reservoirs of VACV, but the risk factor of garbage destination should be a future target of investigation to determinate its role in VACV dissemination.
